# Morphological and histopathological description of *Calyptospora* sp. parasitism in *Cichla monoculus* Spix, 1929 (Osteichthyes, Cichlidae) from the lake region of Pracuúba-Amapá, Brazil

**DOI:** 10.1590/S1984-29612024078

**Published:** 2024-12-20

**Authors:** Eloiza Sarmento Amoras, Jhonata Eduard, Maria do Perpétuo Socorro Progene, José Francisco Berrêdo Reis da Silva, Marcela Nunes Videira, José Ledamir Sindeaux-Neto, Michele Velasco

**Affiliations:** 1 Programa de Pós-graduação em Saúde e Produção Animal na Amazônia - PPGSPAA, Universidade Federal Rural da Amazônia – UFRA, Belém, PA, Brasil; 2 Programa de Pós-graduação em Biologia de Agentes Infecciosos e Parasitários – BAIP, Universidade Federal do Pará – UFPA, Belém, PA, Brasil; 3 Laboratório de Integração Morfo-molecular e Tecnologias – LIMT, Universidade Federal Rural da Amazônia – UFRA, Belém, PA, Brasil; 4 Ciência e Tecnologias de Alimentos – CTA, Universidade Federal Rural da Amazônia – UFRA, Belém, PA Brasil; 5 Museu Paraense Emílio Goeldi, Belém, PA, Brasil; 6 Universidade do Estado do Amapá – UEAP, Macapá, AP, Brasil; 7 Programa de Pós Graduação em Reprodução Animal – REPROAMAZON, Universidade Federal Rural da Amazônia – UFRA, Belém, PA, Brasil

**Keywords:** Coccidia, Tucunaré, Amazon, SEM, Coccídeos, Tucunaré, Amazônia, MEV

## Abstract

The tucunaré (*Cichla* sp.) is an Amazonian fish that is heavily commercialized in the state of Amapá, and it can be infected by a variety of parasites, including coccidia of the genus *Calyptospora*, which are identified at the genus level by analyzing the structures that comprise its morphology. This study aimed to describe the morphology and histopathology of *Calyptospora* sp. parasitism in *Cichla monoculus* Spix, 1929 in the Municipality of Pracuúba, Amapá, Brazil. Nine specimens were acquired from the Lake Sacaizal by artisanal fishermen and transported in isothermal boxes to the Integrated Morpho-molecular and Technologies Laboratory (LIMT) of the Federal Rural University of the Amazon in Belém, Pará, where they were necropsied. Fragments of the liver were removed to visualize cysts using light microscopy and processed for scanning electron microscopy and histology analyses. The analysis revealed that 66.6% of the fish examined had clusters of oocysts in the hepatic region, resulting in the formation of melanomacrophagic centers. The oocysts were sphere-like, with a diameter of 21 µm. They contained four pyriform sporocysts, 8.7 µm long and 4.9 µm wide, with sporopods in the posterior region.

## Resumo

O tucunaré (*Cichla* sp.) é um peixe amazônico de elevada comercialização no Estado do Amapá e que pode estar sujeito a infecção por diferentes parasitos, dentre eles os coccídeos do gênero *Calyptospora* sp. que são identificados quanto ao gênero, por meio da análise das estruturas que compõem sua morfologia. Este trabalho tem o objetivo de realizar a descrição morfológica e histopatológica do parasitismo por *Calyptospora* sp. em *Cichla monoculus* Spix, 1929, oriundos do Município de Pracuúba, Amapá, Brasil. Os 9 espécimes mortos foram adquiridos do lago Sacaizal por pescadores artesanais, em seguida transportados em caixas isotérmicas até o Laboratório de Integração Morfo-molecular e Tecnologias (LIMT) da Universidade Federal Rural da Amazônia, em Belém, Pará, aonde foram necropsiados e retirados fragmentos do fígado para visualização de cistos em microscopia de luz (ML) e processados para microscopia eletrônica de Varredura (MEV) e histologia. Por meio das análises, foi possível confirmar que 66,6% dos peixes analisados apresentavam aglomerados de oocistos na região hepática com a formação de centros melanomacrofágicos. Os oocistos eram arredondados, com diâmetro de 21 µm, contendo quatro esporocistos piriformes de 8.7 µm de comprimento e 4.9 µm de largura, que apresentavam esporopódes na região posterior.

## Introduction

The state of Amapá in the Municipality of Pracuúba has five primary fishing locations, including the “lake region.” This area is known for its rivers and streams, which are home to a diverse range of fish (Silva & Dias, 2010; Zacardi et al., 2021). In this location, fish of the genus *Cichla* Schneider, 1801 are among the most commonly caught by extractive fishing. Species of the genus are generally known in Brazilian territory as “tucunarés.” They are native to the Amazon basin and belong to the family Cichlidae within the order Cichliformes (Kullander, 2003; Kullander & Ferreira, 2006; Zacardi et al., 2021).

*Cichla monoculus* Spix, 1929, is resident in floodplain lakes and other ecotopes in Brazil and Peru. Their diet typically consisted of shrimp and fish. This species is characterized by a horizontal spot on the surface of the pectoral fin and a discontinuous lateral line on both sides (Kullander, 1987; Rabelo & Araújo-Lima, 2002; Neto et al., 2017; Kullander & Ferreira, 2006).

Apicomplexa Levine, 1970, is a protist phylum that includes obligate intracellular parasites found in both vertebrates and invertebrates. The class Coccidia Leuckart, 1897 comprises species that infect the gastrointestinal systems of their hosts. In freshwater fish, the genus *Calyptospora* is notable as the cause of highly prevalent liver infections that can be evident as a change in the color of the liver to whitish (Bonar et al., 2006; Acosta et al., 2016; Eiras et al., 2016; Votýpka et al., 2016; Ramos et al., 2018).

In general, parasites from *Calyptospora* are distinguished by the formation of oocysts or parasitophorous vacuoles that contain four structures known as sporocysts and are covered by a membrane veil. Each sporocyst contained two sporozoites. Scanning electron microscopy (SEM) can be used to view structural details such as sutures and sporopods, which are useful for determining the similarities and differences between morphological structures and are essential for identification (Azevedo et al., 1995; Matos et al., 1999; Oliveira et al*.,* 2021). In addition, histopathological analyses are effective in detecting parasitic damage to tissues (Videira et al., 2013).

The purpose of this study was to describe the morphology and histopathology of *Calyptospora* sp. parasitizing *C. monoculus* in the lake region of the Municipality of Pracuúba-Amapá, Brazil.

## Material and Methods

### Collection of hosts and parasites

**Figure 1:**
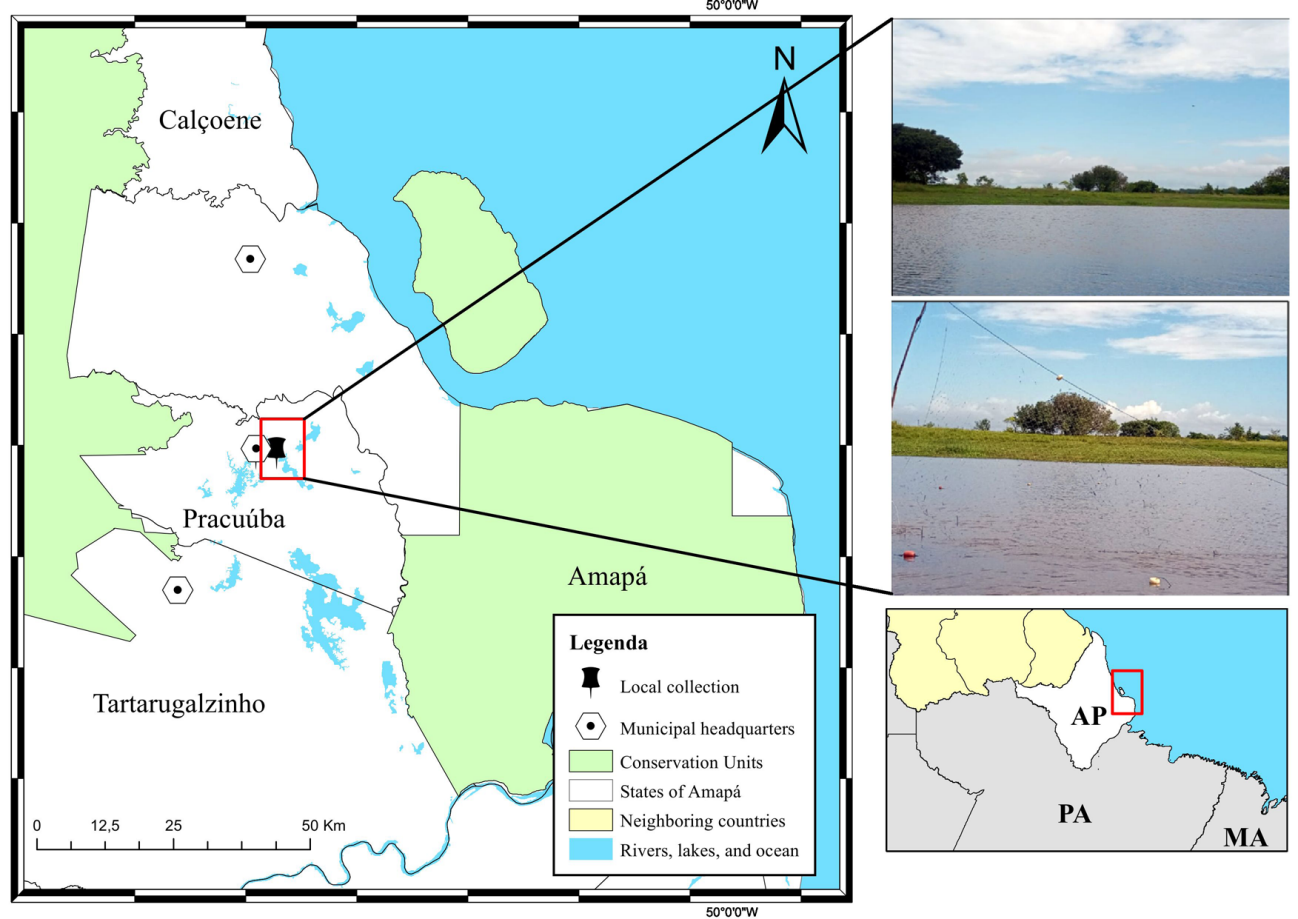
Geographic location map of the fish collection area, in the municipality of Pracuúba, state of Amapá in Brazil.

A total of nine dead specimens of peacock bass (*C. monoculus*) were acquired from artisanal fishermen in the Municipality of Pracuúba-Amapá (1°42'.79”N 50°43'17.5”W), which is close to the towns of Calçoene, Amapá, and Tartarugualzinho (Figure 1). Fish were caught from Lake Sacaizal, packed in isothermal boxes with ice, and transported to the Integrated Morpho-Molecular and Technologies Laboratory (LIMT) of the Federal Rural University of the Amazon (UFRA), Belém *campus* Pará. In the laboratory, specimen biometrics and identification were performed using the dichotomous key proposed by Kullander & Ferreira (2006). The fish were necropsied to inspect the entire body surface, including internal organs, using a Zeiss Stemi DCR stereoscope. Small chunks of the liver with oocysts were extracted, placed in the center of a slide containing a drop of water, softly compressed with a coverslip, and examined using light microscopy.

### Morphometric analysis of oocysts

The morphometric parameters of oocysts (n=17), including oocyst diameter (OD), sporocyst length (SL), and sporocyst width (SW), were measured using ImageJ version 3.2 software as previously described (Schneider et al., 2012). The data were compiled with data from other *Calyptospora* sp. using principal component analysis (PCA) with PAST 3.0 software (Hammer et al., 2001).

### Histopathological analysis

For histological processing, tissue fragments were fixed in Davidson’s solution (95% alcohol, formaldehyde, acetic acid, and distilled water) for 24 h before being dehydrated in an increasing series of alcohol solutions (70%, 80%, 90%, absolute I, absolute II, and absolute III) for 1 h each. After clearing with absolute alcohol–xylol for 30 min, absolute xylol (Xylol I and Xylol II) was successively added for 15 min each. The material was impregnated with paraffin, and the solid blocks that formed were used to obtain 5 μm-thick sections with a model HM315 rotary microtome (Microm). The sections were extruded onto a water bath, collected on glass slides, kept in the oven for 24 h at 60˚C, and stained using hematoxylin-eosin (Luna, 1968).

### SEM

Liver fragments were fixed in 5% glutaraldehyde with 0.2 M sodium cacodylate buffer at pH 7.2 for 3 h at 4°C, washed in the same solution for 2 to 4 h, then fixed in 2% osmium tetroxide in the buffer for 2 h at 4°C. Following the dehydration step, increasing amounts of ethanol were added, followed by critical point drying and coating with gold. Photomicrographs of parasites were acquired at the Scanning Electron Microscopy Laboratory, Museu Paraense Emílio Goeldi. Structural details were observed, and morphometric parameters were measured in mature oocysts (n = 17) using ImageJ 3.2 software. OD, SL, and SW were measured, and average values were determined using Excel 2013 software. The data were compiled for comparison with those of published studies on the morphometry of *Calyptospora* sp.

## Results and Discussion

**Figure 2:**
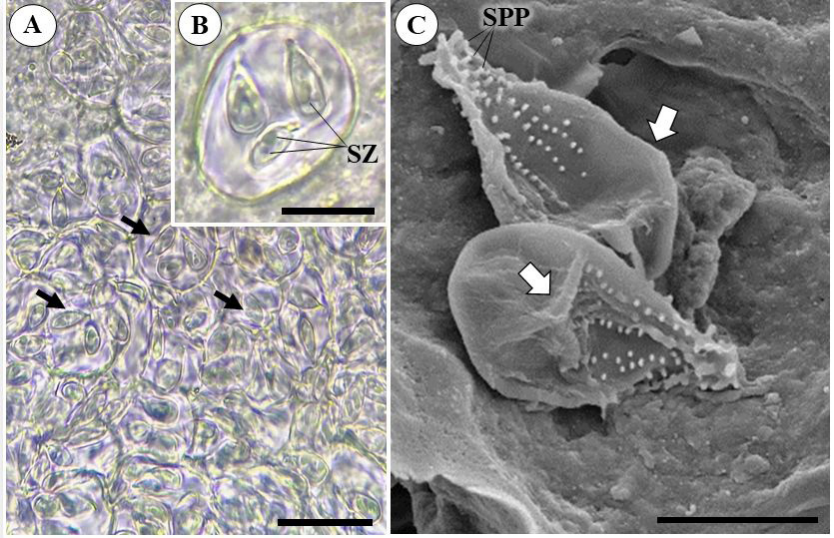
Infection of *Cichla monoculus* Spix, 1929 liver by *Calyptospora* sp. **A**- Groups of *Calyptospora* sp. oocysts, observed under light microscopy (ML) in the hepatic region, highlighting the sporocysts (arrows), Scale Bar 20 μm. **B-** Oocyst with sporocysts, indicating the spozoites internally (SZ), Scale Bar 10 μm. **C-** Sporocysts visualized in greater detail in Scanning Electron Microscopy (SEM), where structures on their surface, the sporopodia (SPP) and the apical and lateral suture lines (arrow) can be observed, Scale Bar 5 μm.

The fish displayed an average weight of 298.0±1.8 g and a length of 21.0±1.2 cm. The prevalence of parasitism by *Calyptospora* sp. was 66.6%, and its morphology was consistent with that of coccidia of the genus. Several mature and rounded oocysts contained pyriform sporocysts in the inner region (Figures 2A-B). SEM of sporocysts revealed externally scattered sporopods (Figure 2C). These are regarded as another crucial characteristic for identification, as documented by Oliveira et al. (2021) in *Serrasalmus rhombeus* (Linnaeus, 1776).

The prevalence of this study was higher than that of 40% of *Calyptospora* sp. in the hepatopancreas of *Cichla temensis* (Humboldt, 1821), reported by Velasco et al. (2012), although there were morphological similarities with the rounded oocysts, pyriform sporocysts and two sporozoites. Santiago et al. (2012) observed parasitism in 56% of *C. temensis* liver specimens from Marajó Island, with a higher incidence during the wet season. Another study reported that fish from the municipality of Vigia in the state of Pará, approximately 60% of *Brachyplatystoma vaillantii* (Valenciennes, 1940) displayed several clustered oocysts that replaced the liver parenchyma region (Silva et al., 2012). Negrão et al. (2019) identified parasitic forms of *Calyptospora* sp. in *Crenicichla lepidota* (Heckel 1840), *Cichla ocelaris* (Bloch & Schneider 1801), *Hoplias malabaricus* (Bloch, 1794), *Mesonauta festivus* (Heckel 1840), *Hoplerythrinus unitaeniatus* (Spix & Agassiz, 1829), *Astronotus ocellatus* (Agassiz, 1831), *Geophagus proximus* (Castelnau, 1855), *Pterophyllum scalare* (Schultze 1823), *Satanoperca jurupari* (Heckel 1840) and *Heros efasciatus* (Heckel, 1840), from the Curiaú-Amapá River floodplain, which infected the gallbladder, liver, and heart. Morphologically, the oocysts were scarcely recognizable.

The oocysts had an average diameter of 21±2.7 μm, while the pyriform-shaped sporocysts had an average length of 8.7±1.3 μm and a width of 4.9±0.7 μm. This *Calyptospora* presented oocyst diameter, shape, length and width of sporocysts similar to those found by Békési & Molnar (1991), Bonar et al. (2006), Santiago et al. (2012) and Silva et al. (2012, 2019, 2020) (Table 1). Furthermore, this oocyst differed from those reported by Albuquerque & Brasil-Sato (2010), who reported ellipsoidal sporocysts and greater oocyst diameter (24.5 μm) and sporocyst length (11.5 μm). Fournie et al. (1985) also reported this sporocyst form; however, the structural morphometric differences did not differ significantly from the morphotype of this study.

**Table 1:** Morphometric comparison of *Calyptospora* spp. described in fish species, with data on oocyst diameter, shape, length and width of sporocysts, host, infection site and location. All data are in micrometers (µm).

**Species**	**Oocyst**			**Sporocyst**			**Host**	**Site of Infection**	**Location**	**Authors**
	**Diameter**		**Shape**		**Length**	**Width**				
*Calyptospora* sp. 1	21.0±2.7		Pyriform		8.7±1.3	4.9±0.7	*Cichla monoculus*	Liver	Amapá, Brazil	Current study
*Calyptospora* sp. 2	21.2		Pyriform		9.2	3.1	*Cichla temensis*	Hepatopancreas	Pará, Brazil	Santiago et al. (2012)
*Calytpospora* sp. 3	24.5		Ellipsoidal		11.5	4.5	*Triportheus chalceus* and *T. guentheri*	Liver and intestine	Minas Gerais, Brazil	Albuquerque & Brasil-Sato (2010)
*Calytpospora* sp. 4	24.3		Pyriform		8.3	3.7	*Cichla ocellaris*	Liver	Ceará, Brazil	Békési & Molnar (1991)
*Calyptospora* sp. 5	20.8		Pyriform		8.9	4.1	*Brachyplatystoma vaillantii*	Liver	Pará, Brazil	Silva et al. (2012)
*C. gonzaguenisis*	19.6±1.4		Pyriform		9.2±0.6	3.9±0.2	*Triportheus angulatus*	Liver	Maranhão, Brazil	Silva et al. (2020)
*C. paranaidji*	22.1±1.5		Pyriform		9.7±0.5	4.6±0.6	*Cichla piquiti*	Liver	Maranhão, Brazil	Silva et al. (2019)
*C. tucunarensis*	19		Pyriform		7	4	*Arapaima gigas*	Liver	Amazonas, Brazil	Bonar et al. (2006)
*C. empristica*	22		Ellipsoidal		9	5.7	*Fundulus notti*	Liver	Mississipi, EUA	Fournie et al. (1985)

**Figure 3:**
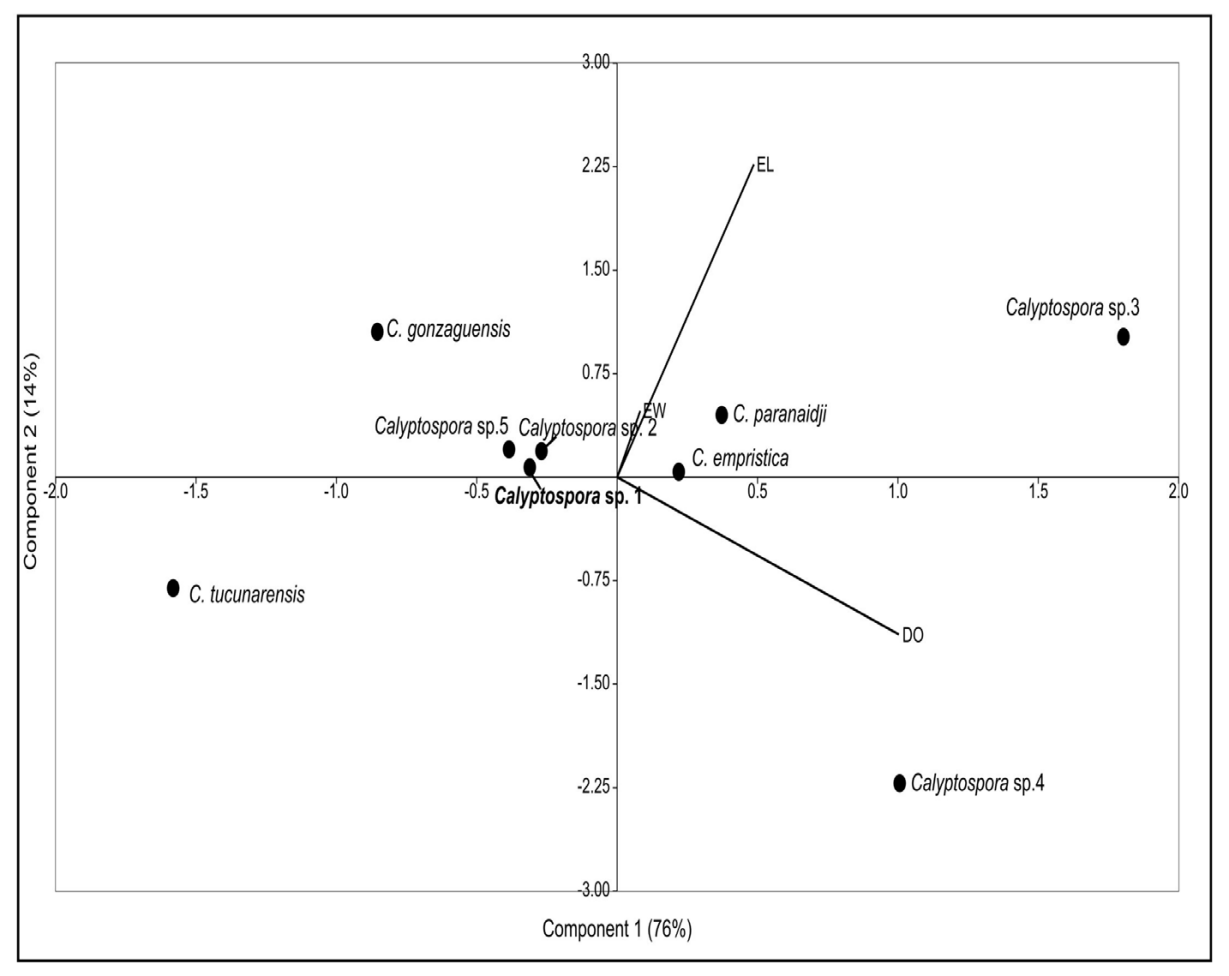
The Principal component analysis (PCA) of oocysts of *Calyptospora* spp. in *Cichla monoculus* from Pracaúuba, Amapá. The oocyst diameter (OD), sporocyst length (SL), and sporocyst width (SW) components were combined. The components 1 and 2, on each axis of the graph, display the variables that vary the most and are in charge of the morphometric ordering of the species.

The morphometric PCA of *Calyptospora* sp. (Figure 3) revealed that component 1 (OD) accounted for approximately 76% of the variance, whereas components 2 and 3 (SL and SW, respectively) accounted for 14% and 10% of the variance, respectively. The findings imply that components 1 and 2 account for 89% of the correlation of variables. The *Calyptospora* sp. oocysts observed in the current study were similar to *Calyptospora* sp. 2 reported in *C. temensis*, with an oocyst diameter of 21.0 *vs.* 21.2 μm, but differed in sporocyst length of 8.7 *vs.* 9.2 μm and sporocyst width 4.9 *vs.* 3.1 μm (Table 1). Although they still demonstrate small morphological variations from *Calyptospora* sp. to *Cichla* sp., it remains uncertain whether they belong to the same taxonomic group. Further molecular data, specifically markers of small ribosomal subunit DNA data, are required (Berto et al., 2014).

**Figure 4:**
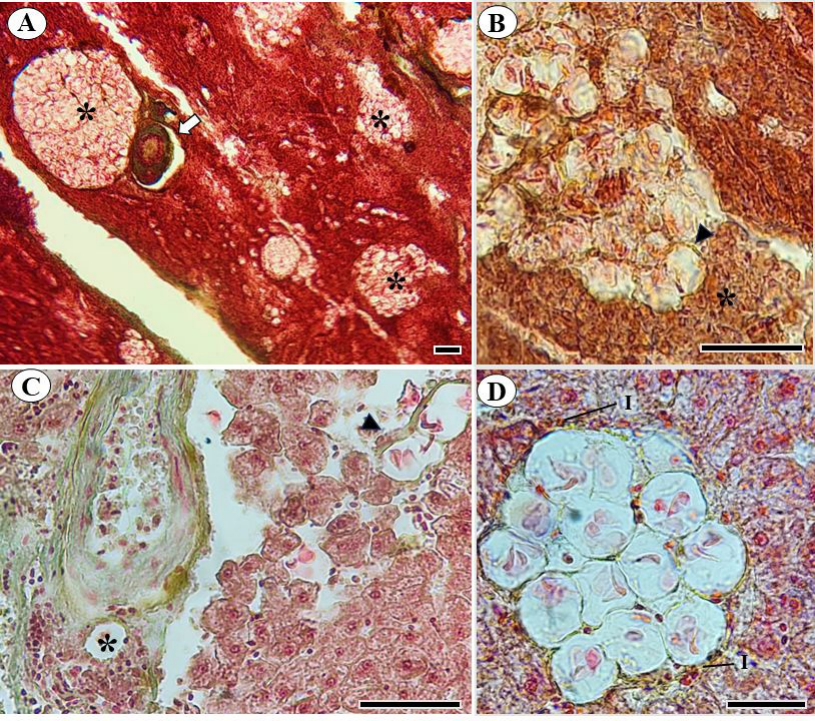
Gomori-stained photomicrographs of *Calyptospora* sp. oocysts in the liver parenchyma of *Cichla monoculus* Spix, 1929. **A**- Oocysts (*) adjacent to the hepatic artery (arrow), Scale Bar 50 μm. **B**- Adjacent oocytes (arrowhead) in a region of hepatopancreas (*), Scale Bar 50 μm. **C**- Oocysts interspersed between hepatocytes with loss of cellular architecture and collagen deposition (arrowhead), perivascular oocysts (*), Scale Bar 50 μm. **D-** Oocysts surrounded by inflammatory infiltrate (I), Scale Bar 20 μm.

According to histopathological analysis, there were many oocysts that matured into hepatocytes and hepatopancreas region; some of these were found near blood arteries with a higher blood supply, which may have been related to the sporocysts' chemotaxis or nutrition (Figure 4 A-C). Furthermore, infiltrates of mononuclear cells were noted, suggesting the presence of an inflammatory process (Figure 4D). Oocysts in the liver parenchyma close to blood vessels were also observed by Oliveira et al. (2021). This location is related to the development to the oocyst stage, where the sporocysts are transported by the blood vessels and mature into oocysts in the hepatocytes, as described in the *C. funduli* life cycle (Fournie et al., 2000). Other studies of *Calyptospora* spp. have shown the presence of inflammatory cells and, in some occasions, necrosis (Fournie & Overstreet, 1993; Békési & Molnár, 1991; Whipps et al., 2012; Videira et al*.,* 2013; Silva et al., 2019; Negrão et al., 2019). This demonstrates that the parasitic intensity found causes damage to the liver tissue of *C. monoculus*.

This is the first report of *Calyptospora* sp. in *C. monoculus*. Morphological and morphometric analyses indicated that parasitism occurred only in the liver tissue of this host. Additional research is required to understand the action of the immunological response and to verify the causes for the formation of melanomacrophagic bodies, the parasite's life cycle, and the forms of prophylaxis and treatment to promote the conservation of the species in its natural habitat.
